# Transcriptomic Analysis of the Negative Effect of Epigallocatechin-3-Gallate from Tea Plant (*Camellia sinensis*) on *Agrobacterium*-Mediated Transformation Efficiency

**DOI:** 10.3390/cimb47030178

**Published:** 2025-03-08

**Authors:** Guizhi Liu, Na Tian, Lan Chen, Siyi Xie, Jinyu Hu, Qifang Jin, Chenyu Shao, Mengdi Huang, Qin Su, Jianan Huang, Zhonghua Liu, Shuoqian Liu

**Affiliations:** 1 Department of Tea Science, College of Horticulture, Hunan Agricultural University, Changsha 410128, China; lgz@stu.hunau.edu.cn (G.L.); tianna5678@163.com (N.T.); lan@stu.hunau.edu.cn (L.C.); siyixie979@stu.hunau.edu.cn (S.X.); hjy225@stu.hunau.edu.cn (J.H.); shaochenyu@stu.hunau.edu.cn (C.S.);; 2 Key Laboratory of Tea Science of Ministry of Education, Hunan Agricultural University, Changsha 410128, China

**Keywords:** transcriptomic, EGCG, *Agrobacterium*-mediated transformation, genetic transformation efficiency, differentially expressed genes (DEGs)

## Abstract

*Agrobacterium*-mediated transformation is a widely used method for plant genetic modification. However, its efficiency in tea plants is notably low, and the underlying molecular mechanisms remain unclear, hindering advancements in the molecular breeding and biology of tea plants. In this study, tobacco was utilized as a model to investigate the effects of various concentrations of epigallocatechin-3-gallate (EGCG) on *Agrobacterium* transformation efficiency. The results demonstrated that at an EGCG concentration of 0.4 mg/mL, *Agrobacterium* nearly lost its ability to transform tobacco. Additionally, malondialdehyde content in *Agrobacterium* was measured before and after EGCG treatment. The findings indicated that EGCG treatment led to an increase in malondialdehyde content. Transcriptome sequencing analysis revealed that differentially expressed genes (DEGs) involved in *Agrobacterium* flagellar synthesis and secretion systems were down-regulated under EGCG stress. Furthermore, *flgE*, *virB4*, and *virB6* were identified as hub genes through weighted gene co-expression network analysis (WGCNA). These results elucidate the dynamic mechanisms by which EGCG affects *Agrobacterium* at both the physicochemical and molecular levels, providing a theoretical basis for optimizing genetic transformation in tea plants.

## 1. Introduction

Tea (*Camellia sinensis* (L.) O. Kuntze) is a widely cultivated and commercially valuable crop. The long growth cycle and self-incompatibility of tea plants limit the effectiveness of conventional hybrid breeding methods for improving tea varieties [1]. Consequently, biotechnological approaches, such as genetic engineering, play a crucial role in tea plant breeding. *Agrobacterium*’s Ti plasmid enables the integration of DNA into plant chromosomes, facilitating synchronous expression with endogenous plant genes at a relatively low cost [2]. Researchers have successfully applied *Agrobacterium*-mediated transformation (AMT) to enhance crops like apple, citrus, and maize [3,4,5]. However, the polyphenolic compounds present in tea exhibit significant bacteriostatic effects. These compounds can kill *Agrobacteriu*, impair bacterial adsorption to plant cells, interfere with *Agrobacterium* chemotaxis, prevent bacteria from reaching plant wounds to cause infection, and inhibit the expression of *Agrobacterium* virulence genes, thus reducing transformation efficiency [1,6,7]. These challenges contribute to the low efficiency of *Agrobacterium*-mediated genetic transformation in tea, making it difficult to study the tea genetic transformation system.

*Agrobacterium*-mediated plant transformation involves a series of complex steps [8]: (1) detecting signal molecules released by plant wounds; (2) *A. tumefaciens* migrating to the injured tissue and attaching to the surface of the host cell in response to these signals; (3) transferring the T-DNA complex to the host cell; and (4) integrating the T-DNA into the host cell genome. Any failure in these steps can impede the transformation process.

The initial step in *Agrobacterium*-mediated plant transformation is the recognition of plant signals and the attachment of *Agrobacterium* to plant cells [9]. During this process, *Agrobacterium* forms a biofilm to stabilize its attachment, thereby enhancing the transformation process [10]. Therefore, biofilm formation is essential for successful plant transformation by *Agrobacterium* [10]. Early observations had indicated that the flagellum and flagellum-based motility contribute to the successful colonization of surfaces [11]. Bacteria swim to plant wounds using their flagella and establish effective contact with host cells [11,12]. In addition, flagella can serve as adhesins and increase the chances of remaining on the surface [11,13]. For example, *Agrobacterium tumefaciens* C58 mutants with a paralyzed flagellum, but flagellum gene mutants, showed strong defects in biofilm formation [10]. A mutant of *Agrobacterium* lacking a complete flagellar structure showed poor attachment to host cell surfaces, even after extended incubation, leading to reduced virulence [14,15]. The potential of epigallocatechin-3-gallate (EGCG) as an effective anti-biofilm agent has been widely acknowledged, with multiple reports highlighting its significant inhibitory effect on bacterial motility and its ability to impede biofilm formation [16,17,18]. Nevertheless, the effects and mechanism of EGCG on the formation of *Agrobacterium* biofilm, as well as its molecular targets, remain largely unknown.

*Agrobacterium*, as a plant pathogen, can transfer T-DNA horizontally, which is integrated into the plant genome through non-homologous recombination [19]. This process is fundamental to *Agrobacterium*-mediated transformation and depends on the bacterial secretion system [20]. *Agrobacterium* conjugation is mediated by the type IV secretion system (T4SS), which comprises 12 proteins (VirB1–VirB11 and VirD4) [21]. Typically, *A. tumefaciens* C58 transfers T-DNA, effector proteins, and virulence genes to plant cells via T4SS machinery in the pTi (tumor-inducer) plasmid [22,23]. The pTi plasmid carries several T4SS genes, including virulence (*vir*), avirulence homolog (*avh*), and Tra system (trb) genes, with *vir* being essential for T-DNA transfer [24]. However, catechins can inhibit the expression of *vir* genes and antagonize various Vir proteins produced by *Agrobacterium*, thereby disrupting the T4SS and hindering T-DNA transfer to tea plant cells, ultimately impairing the transformation process [25].

The insensitivity of tea plants to *Agrobacterium* and the low transformation efficiency remain major challenges in *Agrobacterium*-mediated genetic transformation of tea explants [1]. This is largely attributed to the significant antibacterial effects of polyphenols, such as the flavanols (mainly catechins), anthocyanins, and flavonoids present in tea plants [25,26]. To mitigate the inhibitory effects of polyphenols, various strategies have been employed, including the addition of autoclaved L-glutamine [27], polyphenol adsorbents (vinylpyrrolidone, charcoal) [28], and acetosyringone (AS) [29] to the medium. After high-pressure sterilization, the chemical structure of L-glutamine is altered, producing α-aminoglutarimide. This compound can reduce the bacteriostatic effects of polyphenols [27]. While these methods have somewhat alleviated the inhibitory effects of tea polyphenols on *Agrobacterium*, the genetic transformation system for tea plants still requires further optimization [1].

Previous studies have primarily focused on evaluating the antimicrobial activity of catechins [30,31], while recent research has revealed that tea leaves induce aberrant bacterial cell morphology, thereby impeding successful *A. tumefaciens* transfection [32]. However, among catechins, EGCG stands out as the most abundant and potent polyphenol, accounting for 50–80% of the total catechins in tea leaves. EGCG is characterized by its unique galloyl group at the 3′ position of the flavonoid structure, which contributes to its strong antioxidant and antimicrobial properties [16,17]. However, few studies have explored the ability of EGCG to mitigate the bacterial infection of tea plant cells based on its effect on *Agrobacterium* gene expression. The objectives of this study were as follows: First, we observed the effect of different concentrations of EGCG on the transformation efficiency of *Agrobacterium* using microscopy. Additionally, we investigated the effects of EGCG on *Agrobacterium* by measuring malondialdehyde (MDA) content before and after EGCG treatment. To understand the molecular mechanisms behind the low *Agrobacterium*-mediated transformation (AMT) efficiency in tea plants caused by EGCG, transcriptomic analysis was employed to examine the expression profiles of *Agrobacterium*’s differentially expressed genes (DEGs) under EGCG stress. Key genes were identified using KEGG annotation and weighted gene co-expression network analysis (WGCNA).

## 2. Materials and Methods

### 2.1. Plant Materials and Sample Collection

The tobacco materials used in this study were sterile seedlings, which were inoculated into MS solid medium after the disinfection of *Nicotiana benthamiana* seeds. The tobacco plants were grown for 4 weeks for subsequent fluorescence observation experiments.

In this study, the empty vector pSuper-1300-GFP, which solely encodes the GFP protein, was introduced into tobacco cells, and subsequent fluorescence observations were conducted using microscopy [33].

*Agrobacterium tumefaciens* GV3101 was inoculated in LB medium overnight in the dark (28 °C, 200 rpm). The overnight culture was centrifuged at 5000 rpm for 15 min to collect the bacteria, which were then resuspended in a solution containing 1 mM magnesium chloride, 1 mM MES, 200 μM AS, and sterile water to a final volume of 200 mL. The resulting GV3101 infection solution was divided into two groups: the control group without EGCG and the treatment group with 0.4 mg/mL of EGCG added. Both *Agrobacterium* infection solutions were placed in the same incubator simultaneously and incubated at 28 °C for 0, 2, 7, and 15 h. After incubation, the infection solution was transferred to a centrifuge tube and centrifuged at 7500 rpm and 4 °C for 5 min to obtain the bacterial precipitate. The bacterial precipitate was resuspended in sterile water and centrifuged again at 7500 rpm and 4 °C for 10 min to obtain the *Agrobacterium* precipitate, which was immediately frozen in liquid nitrogen for transcriptome analysis. Three biological replicates were prepared for each sample group at each time point.

### 2.2. Green Fluorescence GFP Observation

The GFP-labeled 1300 vector (pSuper-1300-GFP) was transferred into GV3101, which was then inoculated in LB liquid medium overnight at 28 °C and 200 rpm in the dark. Tobacco leaves were transformed using the infiltration method, with the plants watered the day before to keep the leaves fresh. After inoculation, the tobacco was cultured in darkness for 48 h, followed by a 16 h/8 h light/dark cycle for another 48 h at 25 °C and 74% relative humidity. The transformed tobacco was observed under a Zeiss upright fluorescence microscope (Axio Scope A1, Zeiss, Jena, Germany).

### 2.3. Measurement of MDA

The obtained GV3101 infection solution was divided into two groups. The control group was not treated with EGCG and was cultured at 28 °C for 2, 7, and 15 h. The treatment group was administered 0.4 mg/mL EGCG and cultured at 28 °C for 2, 7, and 15 h. The treated bacterial solution was analyzed using a Solarbio kit, and MDA levels were determined colorimetrically according to the manufacturer’s instructions.

MDA levels were measured at three time points (2, 7, and 15 h) with three technical replicates per time point. The data were analyzed using repeated measures ANOVA followed by Bonferroni’s post hoc test to compare differences between time points. Statistical significance was set at *p* < 0.05. All analyses were performed using GraphPad Prism 9.

### 2.4. RNA Extraction and Transcriptome Sequencing

The sequencing library was constructed using the NEBNext Q5 Hot Start HiFi PCR Master Mix kit (NEB, Massachusetts, United States) and sequenced on the Illumina Novaseq 6000 platform. Raw sequencing data were subjected to quality control using FastQC (version 0.11.9) and trimmed using Trimmomatic (version 0.39) to remove low-quality reads and adapters. Clean reads were then aligned to the *Agrobacterium tumefaciens* strain C58 genome [34] using Bowtie2 (version 2.4.2), a widely used and reliable tool for short-read alignment.

### 2.5. Gene Annotation, Enrichment, and Differential Expression Analysis

Functional annotation of genes was based on publicly available annotations of *Agrobacterium tumefaciens* strain C58 genome in databases including GenBank Nonredundant, Protein family (Pfam), Swiss-Prot, Eukaryotic Ortholog Groups (KOGs), Gene Ontology (GO), and Kyoto Encyclopedia of Genes and Genomes (KEGG). Differential gene analysis involved mapping functional classifications using GO and KEGG annotations. Over-represented GO terms and KEGG pathways were identified using false discovery rate (FDR) calculations, with a threshold of FDR ≤ 0.05 for significant enrichment. 

### 2.6. Co-Expression Network Analysis

WGCNA was conducted using the R package WGCNA (v3.6.0) [35]. Genes with FPKM > 1 and coefficient of variation (CV) > 0.5 were retained to ensure reliable co-expression networks, excluding lowly expressed and low-variability genes. The analysis parameters included a similarity threshold of 0.5 for module fusion, a minimum module size of 50, a soft threshold power of 14 (based on scale-free topology fit and mean connectivity), and a minimum height of 0.05 for merging modules. Co-expression networks were visualized in Cytoscape (v3.9.0), focusing on the top 200 gene pairs in each significant module to highlight key interactions [36].

### 2.7. Quantitative RT-PCR (qRT-PCR) Validation

To confirm the RNA sequencing results, twelve genes (*motA*, *flgB*, *flaA*, *flab*, *exoY*, *kdpD*, *kdpE*, *virB10*, *virB11*, *virD4*, *attM*, *and traG*) were selected for qRT-PCR analysis, and cDNA was synthesized using the PrimeScriptTM RT Reagent Kit (Takara, Dalian, China). qRT-PCR assays were performed on a quantistudio 3 Real-Time PCR system (Applied Biosystems, Carlsbad, CA, USA) using the TB Green^TM^ Premix Ex Taq^TM^ II kit (Takara, Dalian, China). All primers for qRT-PCR analysis were designed using the automated primer design tools in the primer Premier (v5.0) software (Premier Biosoft International, Palo Alto, CA, USA) and DNAMAN (v8.0) software (Lynnon Biosoft, Quebec, Canada). Relative gene expression was normalized using two commonly used internal reference genes, *gyrB* (atu0012) and *dnaC* (atu1084) [32]. All the primer information is listed in (Table S7).

## 3. Results

### 3.1. Effect of EGCG on Agrobacterium-Mediated Genetic Transformation

**Figure 1:**
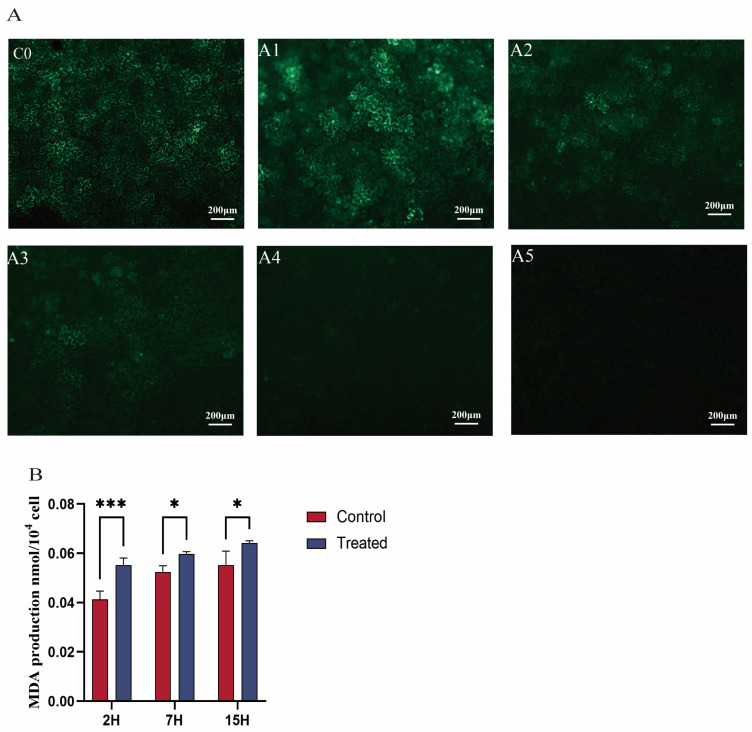
(**A**) Effect of EGCG on the efficiency of *Agrobacterium*-mediated transformation of tobacco. The infection solution of the C0 group did not contain EGCG; EGCG was added to the infection solution of the A1–A5 groups, and the concentration was 0.1, 0.2, 0.3, 0.4, and 0.5 mg/mL, respectively. (**B**) Effect of EGCG on the MDA content in *Agrobacterium tumefaciens*. * indicates that *p* < 0.05; *** represents significant differences when *p* < 0.001.

Microscopic fluorescence images revealed that fluorescence expression in tobacco leaf cells exposed to 0.1 mg/mL EGCG (A1 group) was comparable to that in cells expressing GFP fluorescence without EGCG (C0 group). Both the cytoplasm and nucleus of tobacco leaf epidermal cells emitted clear visibility of GFP under microscopy (Figure 1), indicating the successful transformation of the fluorescent vector into tobacco leaves [33]. In contrast, fluorescence intensity in cells treated with 0.2 mg/mL and 0.3 mg/mL EGCG (A2 and A3 groups) was significantly reduced compared to the C0 group. At an EGCG concentration of 0.4 mg/mL, fluorescence expression was completely absent in the tobacco leaf cells (Figure 1A). Additionally, tea plant cells successfully transformed by *Agrobacterium* exhibited intense and vivid green fluorescence under microscopy, with increased fluorescence intensity and larger luminous areas indicating enhanced transformation efficacy [33]. In conclusion, these results demonstrate that EGCG inhibits *Agrobacterium*-mediated transformation, with near-complete inhibition observed at an EGCG concentration of 0.4 mg/mL.

### 3.2. Effect of EGCG on the Agrobacterium Cell Membrane

*Agrobacterium* transformation requires intercellular recognition through glycoproteins on the cell membrane. To further confirm the effect of EGCG on *A*. transformation, this study measured the malondialdehyde (MDA) content in bacteria. The results showed that the MDA content in *Agrobacterium* treated with EGCG (0.4 mg/mL) was significantly higher than that in the control group (Figure 1B). In the control group, MDA content increased gradually over time. The MDA content in the treatment group increased by 33.4% compared with the control group at 2 h, by 14% at 7 h, and by 16.4% at 15 h. In the treatment group, MDA content increased significantly over time, reaching a peak at 15 h after EGCG treatment. In the treatment group, MDA content increased significantly over time, reaching a peak at 15 h after EGCG treatment. As a common indicator of membrane lipid peroxidation, higher MDA content indicates more severe degradation of cell membrane lipids [37]. Therefore, this study demonstrates that EGCG can damage the cell membrane of *Agrobacterium* and hinder transformation.

### 3.3. Differential Gene Expression Analysis in Agrobacterium Caused by EGCG

In this study, the control groups cultured for 0, 2, 7, and 15 h without EGCG treatment were labeled as C2, C7, and C15, respectively. Similarly, the treatment groups cultured for 2, 7, and 15 h with 0.4 mg/mL EGCG were labeled as T2, T7, and T15. Using total RNA from C0, C2, C7, C15, T2, T7, and T15 (for Table S1, we constructed and sequenced 21 cDNA libraries via Illumina sequencing. Each library generated an average of 1.95 Gb of clean bases with an average Q30 level of 89.7% (Table S1)). The comparison rate with the reference genome ranged from 84.92% to 94.06% per sample. The number of valid reads obtained after filtering out low-quality bases ranged from 12,029,836 to 16,302,662, with an average comparison rate of 92.4%. The dataset was deposited at NCBI under the biological project number PRJNA1067299.

**Figure 2:**
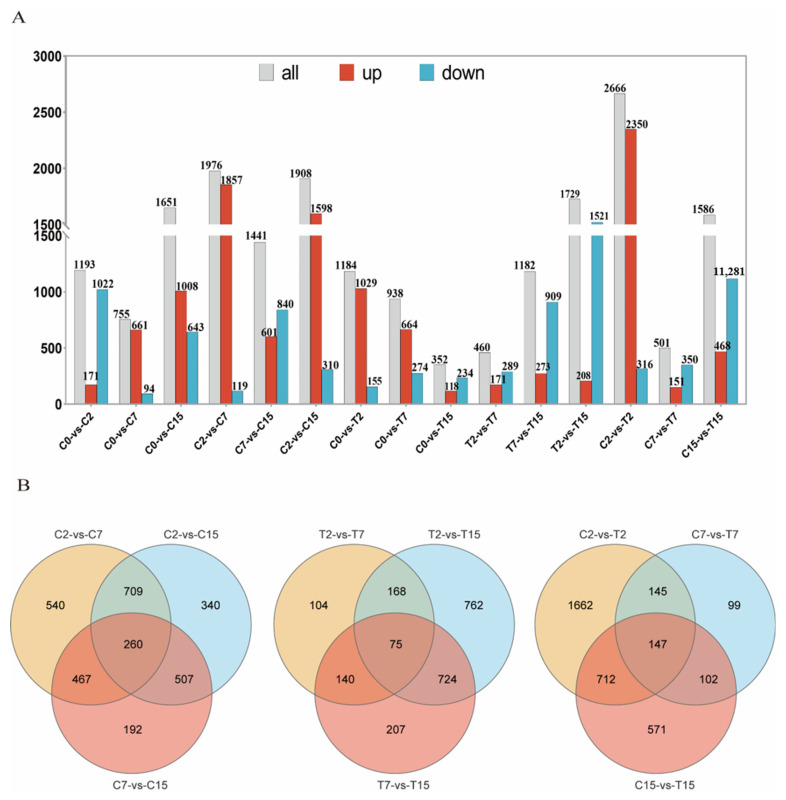
The RNA-seq data expression pattern of the samples. (**A**) The number of up-regulated and down-regulated DEGs in different combinations. (**B**) Pairwise comparison of co-expression and the sole expression of DGEs as a Venn diagram.

**Figure 3:**
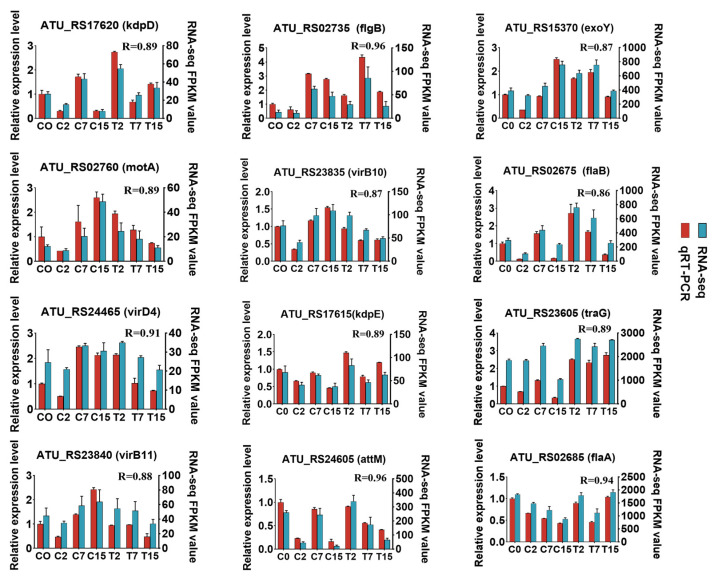
Quantitative RT-PCR validation. Twelve DEGs were selected for qRT-PCR detection. Error bars indicate the standard deviation of the three independent replicates.

In this study, 5031 *Agrobacterium* genes were further identified, of which 4024 were differentially expressed in 15 two-by-two comparisons. The following comparisons were analyzed: 2666 DEGs for the C2 and T2 comparison (2350 up-regulated and 316 down-regulated), 501 for the C7 and T7 comparison (151 up-regulated and 350 down-regulated), and 1586 for the C15 and T15 comparison (468 up-regulated and 1118 down-regulated) (Figure 2A). To validate the differential expression patterns observed in the transcriptomic data, 12 genes were randomly selected for qRT-PCR analysis (Figure 3). The qRT-PCR results showed that the expression changes of the selected genes were consistent with the RNA-seq data, with correlation coefficients (R) all above 0.85. These results confirmed the reliability and accuracy of the transcriptomic data in this study.

### 3.4. Venn Analysis of the DEGs

Venn diagram analysis was used to identify the *Agrobacterium tumefaciens* genes that may be affected by EGCG during the infestation of tea pants. The diagram shows the number of specific and overlapping DEGs (Figure 2B). In the three two-by-two comparisons of C2 with C7, C7 with C15, and C2 with C15, a total of 260 common genes were observed. In the two-by-two comparisons of T2 with T7, T7 with T15, and T2 with T15, 75 common genes were identified. Finally, in the two-by-two comparisons of C2 with T2, C7 with T7, and C15 with T15, 147 common genes were found (Figure 2B). After removing duplicate DEGs from the three comparisons, 432 DEGs were selected. In the control group (C2, C7, C15), we identified 260 common DEGs reflecting natural physiological changes over time. These genes were included in the Venn diagram analysis to ensure that the final list of 432 key genes was not biased by natural variations.

We performed trend analysis on the 432 genes obtained, and 4 significantly enriched modules were identified the treatment group (Figure S1A). The number of genes in profile 16 was the largest, with a total of 120, and the gene expression trend of this module was up-regulated and then down-regulated. There were 83 genes in profile 13, and the module genes showed a trend of up-regulation and then down-regulation. The genes in these two modules are mainly involved in bacterial ribosomes, two-component systems, quorum sensing, biofilm formation, and flagellar synthesis (Table S2A, B). The two-component system plays an important role in the process of bacteria sensing and responding to changes in the external environment. It can detect external stimulus signals and participate in regulating important processes such as bacterial cell division, virulence, quorum sensing, and biofilm formation [38].

In the control group, we identified five significantly enriched modules (Figure S1B), with profile 6 containing the largest number of genes, totaling 116. The genes in this profile were down-regulated first and then up-regulated immediately, and they are mainly involved in bacterial quorum sensing and ribosome synthesis (Table S3).

Combined with the results of trend analysis, we found that EGCG inhibited most of the genes in *Agrobacterium*. The main inhibited genes were involved in bacterial motility, signal transduction, and ribosome synthesis.

### 3.5. GO Enrichment Analysis of the DEGs

**Figure 4:**
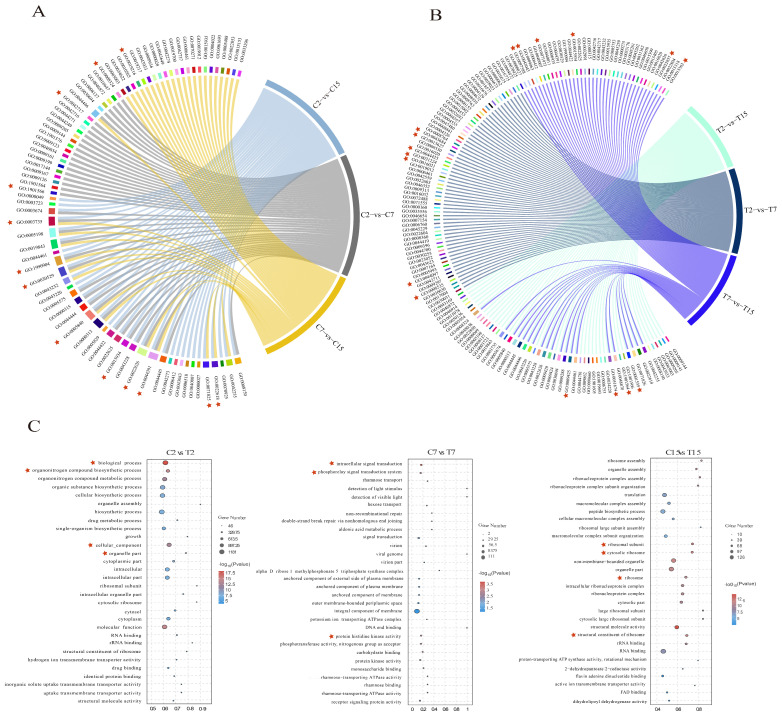
GO enrichment analysis of DEGs. (**A**) Overall enrichment terms of DEGs of C2 vs. C7, C7 vs. C15, and C2 vs. C15 comparisons. (**B**) Overall enrichment terms of DEGs of T2 vs. T7, T7 vs. T15, and T2 vs. T15 comparisons. (**C**) The top 10 significantly enriched GO terms of BP, CC, and MF categories of the C2 vs. T2, C7 vs. T7, and C15 vs. T15 comparisons. The asterisk represents the significantly enriched GO terms in the comparison group. Red asterisks mark the key enriched pathways in each comparison group.

In this study, GO enrichment analysis was performed on the differential genes in each comparison group to explore changes in the *Agrobacterium* infection process under the influence of EGCG. In the control group, 41, 36, and 35 GO terms were significantly enriched in the C2 vs. C7, C7 vs. C15, and C2 vs. C15 comparisons, respectively (Table S4A). For the C2 vs. C7 comparison, the enriched GO terms included the BP (biological process) classes of biosynthesis of organic nitrogen compounds (GO:1901566) and cytosolic biosynthetic process of nitrogen compounds (GO:0044271). The CC (cellular component) classes included the synthesis of ribosomal subunits (GO:0044391), cytoplasmic ribosomes (GO:0022626), macroribosomal subunits (GO:0015934), and ribosomes (GO:0005840). The MF (molecular function) class was significantly enriched for cation transmembrane transporter protein activity (GO:0008324). Additionally, significant enrichment was observed in four GO terms: lipid metabolic processes (GO:0019216), ribonucleoprotein complex assembly (GO:0022618), intracellular ribonucleoprotein complex (GO:0030529), and ribonucleoprotein complex (GO:1990904) (Figure 4A and Table S4A).

When comparing C2 and C15, the GO terms identified were GO:0022618 for ribonucleoprotein complex assembly, GO:0071826 for ribonucleoprotein complex subunit organization, GO:0006518 for peptide metabolic processes, GO:0044391 for ribosomal subunits, GO:0015934 for large ribosomal subunits, and GO:0003735 for the structural components of ribosomes. The control group displayed 19 terms related to ribosome synthesis, 13 terms related to ion transport, and 5 terms related to lipid metabolism (Figure 4A and Table S4A).

In the EGCG-treated group, 46, 50, and 44 GO terms were significantly enriched in the T2 versus T7, T7 versus T15, and T2 versus T15 comparisons, respectively (Table S4B). In addition, respiratory chain complex IV assembly (GO:0008535), type IV secretion system secretion (GO:0044097), and signal transduction (GO:0007165), as well as components of the membrane (GO: 0016021), intrinsic components of the membrane (GO:0031224), and membrane fractions (GO:0044425), were significantly enriched in the comparisons of T2 versus T7, while the type II protein secretion system complex (GO:0015627) and type IV secretion system complex (GO:0043684) may be affected by EGCG. In the comparison of T7 and T15, bacterial-type flagellum-dependent cell motility (GO:0097588), peptide biosynthesis process (GO:0043043), bacterial-type flagellum-dependent cell motility(GO:0071973), cilia- or flagellum-dependent cell motility (GO:0001534), cellular localization (GO:0051674), transmembrane transporter protein activity (GO:0022857), and uptake transmembrane transporter protein activity (GO:0015563) may be affected by EGCG. Cilia- or flagellum-dependent cell motility (GO:0001539), organic nitrogen compound metabolic processes (GO:1901564), and bacterial-type flagellar basal bodies (GO:0009425) were significantly enriched in the comparison of T2 and T15. In the treatment group, there were 6 GO terms related to ribosome synthesis, 15 GO terms related to biofilm, and 19 GO terms related to bacterial motility (Figure 4B and Table S4B), which may be affected by EGCG. Enrichment of ribosome synthesis and the lipid–nitrogen metabolism processes was reduced in the EGCG-treated group compared with the control group; biofilm synthesis, bacterial motility, and localization processes were enriched, and these processes may be responsible for the interference of EGCG with *Agrobacterium* infestation. It is noteworthy that following EGCG stress treatment of *Agrobacterium*, there was an enrichment of processes related to biofilm synthesis, bacterial motility, and localization. *A. tumefaciens* is capable of sensing chemical signals released from plant wounds and utilizing its swimming motility to reach these sites, thereby enhancing its own virulence through the formation of biofilms [12]. The GO enrichment results demonstrated that *Agrobacterium* infection of tea leaves was challenging, likely due to interference with its membrane formation and motility.

The top 10 BP, CC, and MF categories of GO annotations during *Agrobacterium* development after EGCG treatment were further analyzed, including C2 compared with T2, C7 compared with T7, and C15 compared with T15 (Figure 4C). As can be seen from the C2 and T2 comparisons, EGCG treatment for 2 h significantly enriched GO terms mainly related to cellular structures, with significant enrichment in biological processes (GO:0008150), organic nitrogen compound biosynthesis processes (GO:1901566), cellular component processes (GO:0005575), and organelle parts (GO:0044422). After EGCG treatment for 7 h, the significantly enriched GO terms were mainly related to signal transduction processes, with significant enrichment of intracellular signal transduction (GO:0035556), the phosphorelay signal transduction system (GO:0000160), and protein histidine kinase activity (GO:0004673). After EGCG treatment for 15 h, ribosomal synthesis was significantly affected, with structural molecular activity (GO:0005198), the cytoplasmic ribosome (GO:0022626), the ribosomal subunit (GO:0044391), and the ribosome (GO:0005840) also being significantly enriched. In summary, the difficulty experienced by *Agrobacterium* in infesting tea pants may be related to the process of nitrogen metabolism, ion transport process, biofilm synthesis, flagellar synthesis, and ribosome synthesis.

In summary, nitrogen metabolism, ion transport, biofilm synthesis, flagellar synthesis, ribosome synthesis, and other metabolic processes are more active under EGCG stress. The growth of *Agrobacterium* requires a variety of nutrients, such as nitrogen, peptides, zinc, potassium, etc. [32]. By analyzing the GO enrichment of each group according to the treatment time, we found that EGCG may first cause the abnormal growth of bacteria by interfering with the structural composition of *Agrobacterium* and then disrupts the signal transduction process of bacteria, affects the activity of bacteria, and hinders the transformation process.

### 3.6. KEGG Enrichment Analysis

To characterize the bacterial pathways affected by EGCG in *Agrobacterium*, in this study, KEGG enrichment analysis was performed on the differential genes in each comparative group, and the top 30 enriched pathways were employed to plot the enrichment circle diagram (Figure S2).

A total of 126 KEGG pathways were enriched in T2 vs. C2, including 22 significant terms (*p* < 0.05). These included the ribosome (ko03010), the flagellar assembly pathway (ko02040), aminoacyl-tRNA biosynthesis (ko00970), oxidative phosphorylation (ko00190), and thiamine metabolism (ko00730) (Table S5A).

A total of 76 KEGG pathways were enriched in C7 vs. T7, including 5 significant terms (*p* < 0.05). These included the ABC transporter protein pathway (ko02010), non-homologous end joining (ko03450), biotin metabolism (ko00780), ascorbic acid and aldolate metabolism (ko00053), and glutathione metabolism (ko00480) (Table S5B).

A total of 115 KEGG pathways were enriched in C15 vs. T15, including 8 significant terms (*p* < 0.05). These included the ribosomes (ko03010), flagellar assembly pathway (ko02040), group sensing effect (ko02024), cyanoamino acid metabolism (ko00460), and RNA degradation (ko03018) (Table S5C).

We found that the flagellar assembly of *Agrobacterium* was significantly affected by EGCG. In addition, the bacterial secretion system pathway (ko03070) was enriched at 2 and 15 h (Table S5). Although these pathways are not included in the first few enrichments, the number of enriched genes is large, so it is worth paying attention to. Based on a previous study of the *Agrobacterium* transformation process [19], combined with the significance of differential genes in this study and the proportion of total genes in this metabolic pathway, the bacterial flagellar assembly pathway (ko02040) and secretory system pathway (ko03070) will be analyzed below.

### 3.7. The DEGs Involved in the Flagellar Synthesis Pathway

**Figure 5:**
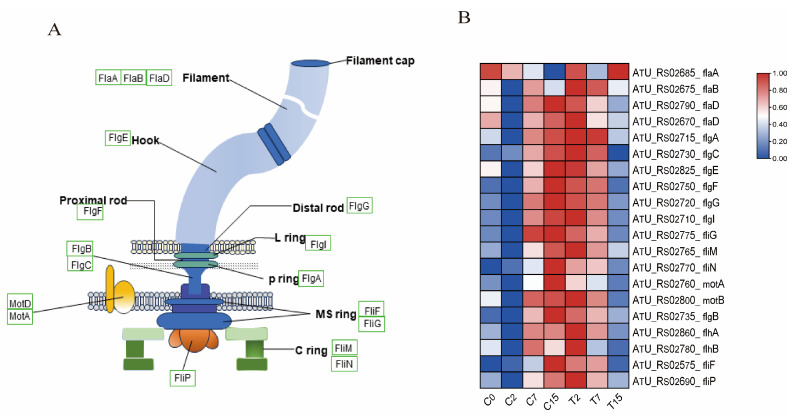
Bacterial flagellar synthesis pathway and related gene expression. (**A**) Bacterial flagellar structure diagram, where green boxes indicate the identified genes. (**B**) The expression levels of DEGs and DEGs related to the flagellar synthesis pathway were normalized by log2 (FPKM).

The flagellum is an organelle involved in bacterial movement and is composed of thousands of protein subunits. In this study, 20 differential genes were identified in the flagellar assembly pathway(ko02040) (Figure 5A,B and Table S6A), including 4 *fla* genes, 7 *flg* genes, 2 *flh* genes, 5 *fli* genes, and 2 *mot* genes. Compared with the control group, the expression of one gene was down-regulated after 2 h of EGCG treatment. After 7 h, nine genes, including *flaA*, *flaB*, and *flaD*, were down-regulated. After 15 h, 19 genes were down-regulated. This indicates that the motility of *Agrobacterium* is inhibited under EGCG stress, which may hinder the infection of tea leaves by *Agrobacterium.*

### 3.8. The DEGs Involved in the Bacterial Secretion System Pathway

**Figure 6:**
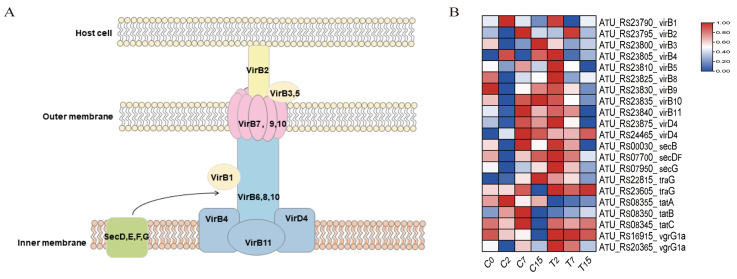
Bacterial secretion system pathway and related genes. (**A**) The structure of the bacterial T4SS secretion system. (**B**) DEGs of the bacterial secretion system pathway, normalizing the expression level of DEGs with log2 (FPKM).

The secretion system of *Agrobacterium* is imperative for plant transformation. A total of 21 genes were identified in the bacterial secretion system pathway (ko03070) (Figure 6, Table S6B). Among these, 13 genes (*virB1-virB11*, *virD4*) were associated with the T4SS system, and 2 genes (*vgrG1a*) were associated with the T6SS system. Six genes (*secB*, *secDF*, *secG*, *traG*, *tatA*, *tatB*) were related to the transmembrane transport of secretory proteins. Compared to the control group, the number of down-regulated genes in the bacterial secretion system increased with the duration of EGCG treatment, with 1, 10, and 11 down-regulated genes observed at 2, 7, and 15 h, respectively.

Compared with the control C0 group, gene expression was severely inhibited at 7 h of treatment. This indicates that the secretion of virulence factors by *Agrobacterium* is impaired under EGCG stress.

### 3.9. WGCNA Analysis

**Figure 7:**
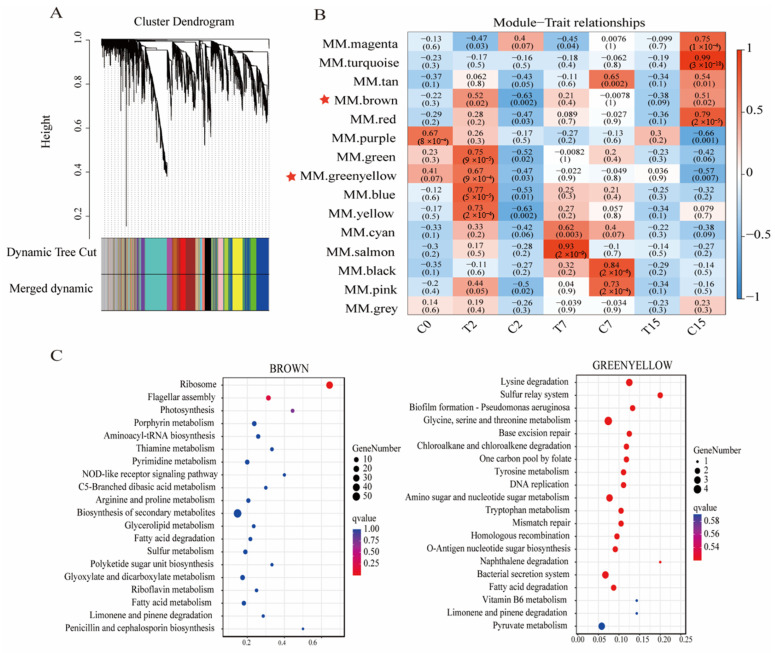
Cluster dendrogram and gene modules after WGCNA analysis. (**A**) Module hierarchical clustering tree based on WGCNA analysis. (**B**) Gene module and sample correlation. Red stars mark key modules. (**C**) Brown and yellow-green modules’ top 20 KEGG enrichment bubble map.

This paper presents the results of a study on the set of genes related to *Agrobacterium* under EGCG stress, based on 5156 genes sequences and phenotypic data. This study generated 15 WGCNA co-expression modules (Figure 7A), labeled with different colors, each containing highly interconnected gene clusters with high correlation coefficients between genes in the same cluster. The number of genes varied across the different modules, with the turquoise module having the highest number, at 790, and the cyan module having the lowest, at 68.

The genes in the 15 modules were correlated with all samples. The darker the color, the stronger the correlation, and a significance level of *p* < 0.02 was used as the standard for screening (Figure 7B). The results showed that the genes of the brown, green, yellow-green, blue, and yellow modules were significantly positively correlated with the T2 group. The genes of the comprehensive color and yellow module were significantly negatively correlated with the C2 group. The gene of the salmon-color module was significantly positively correlated with the T7 group. The genes of the black and pink modules were significantly positively correlated with the C7 group. The genes of the turquoise and red modules were significantly positively correlated with the C15 group.

Then, KEGG enrichment analysis was performed on the genes of the above modules (Figure 7C). We found that the ribosomal pathway (ko03010) and flagellar assembly pathway (ko02040) were significantly enriched in the brown module, while the bacterial secretion system pathway (ko03070) was significantly enriched in the green-yellow module. These pathways are crucial to the *Agrobacterium* transformation process. Therefore, this study focused on the analysis of the genes of the brown and green-yellow modules. We selected the top 200 genes with the highest connectivity to construct a co-expression network and selected the top 20 genes with the highest connectivity as the key hub genes.

**Figure 8:**
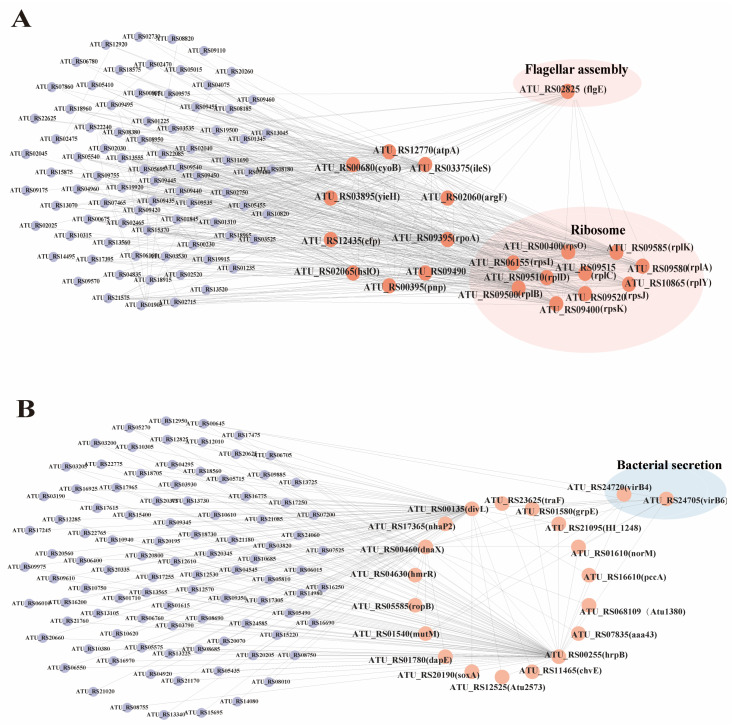
Gene co-expression network analysis. (**A**) Brown module candidate gene co-expression network diagram. (**B**) Yellow-green module candidate gene co-expression network diagram.

The brown module was identified to have 20 hub genes, including *pnp*, *atpA*, *cyoB*, *argF*, *ileS*, *yieH*, *rpoA*, *efp*, and *hslO*. Additionally, 10 genes associated with ribosome synthesis were identified: *rpsO*, *rpsI*, *rpsK*, *rplB*, *rplD*, *rplC*, *rpsJ*, *rplA*, *rplK*, and *rplY*. Finally, one gene related to flagellar synthesis, *flgE*, was also identified (Figure 8A). The yellow-green module hub genes that were included in this study were *divL*, *hrpB*, *dnaX*, *mutM*, *grpE*, *hmrR*, *ropB*, *Atu1380*, *caa43*, *norM*, *chvE*, *Atu2573*, *pccA*, *nhaP2*, *soxA*, *traF*, *HI_1248*, and *dapE*. Additionally, two secretion-system-associated genes, *virB4* and *virB6*, were identified (Figure 8B).

## 4. Discussion

The establishment of a genetic transformation system for the tea plant is of great significance for the study of gene function, secondary metabolite synthesis, and precision breeding of tea plants [1]. *Agrobacterium*-mediated genetic transformation is a widely used tool for validating gene function across various plant species. However, the unique challenges posed by tea polyphenols, particularly catechins, present significant obstacles to successful transformation in tea explants. These polyphenols exert bacteriostatic effects that make tea plants inherently resistant to *Agrobacterium*-mediated transformation [25]. Despite numerous attempts over the years to optimize the genetic transformation system for tea plants, achieving an efficient and reliable transformation protocol remains a significant challenge [1,27,28,29]. In this study, it was found that 0.4 mg/mL EGCG significantly inhibited the transformation efficiency of *Agrobacterium*. Transcriptome analysis of *Agrobacterium* under EGCG treatment revealed that flagellar synthesis and the secretion system were key pathways affected during the transformation process. These findings provide a molecular-level understanding of how EGCG influences *Agrobacterium*, offering a theoretical basis for optimizing genetic transformation in tea plants.

### 4.1. EGCG Is an Important Factor in Tea Plant Recalcitrance to Agrobacterium-Mediated Transformation

Catechins, as the main component of polyphenols, have been reported to reduce transformation efficiency by inhibiting the growth of *Agrobacterium* and reducing the expression of virulence genes [39,40]. Among these catechins, EGCG is particularly potent due to its unique molecular structure, which confers stronger antibacterial activity compared to other catechins such as EGC, ECG, and EC [40]. We compared the effects of different concentrations of EGCG on the efficiency of *Agrobacterium*-mediated transformation in tobacco. The results showed that EGCG inhibited the transformation ability of *Agrobacterium*, with inhibition increasing with higher concentrations (Figure 1A). This result is consistent with findings reported by Song et al. [41], who identified catechins as key components affecting *Agrobacterium*-mediated transformation in tea plants. They found that 6.5 mg/mL catechins had a strong inhibitory effect on *Agrobacterium*. Additionally, they noted that EGCG in fresh tea leaves accounted for 18.3% of total catechins (6.5 mg/mL). In our experiment, the concentration of EGCG was much lower than that in tea leaves, yet we found that at 0.4 mg/mL, EGCG-treated *Agrobacterium* almost completely lost its ability to transform tobacco (Figure 1A). This demonstrates that EGCG has a strong inhibitory effect on *Agrobacterium* transformation efficiency.

It is known that the transformation process of *Agrobacterium* requires intercellular recognition via glycoproteins on the cell membrane [42]. MDA is the end product of membrane lipid peroxidation and serves as an important indicator of the degree of damage to bacterial cells and cell membranes [43]. To further explore the effect of EGCG on the transformation efficiency of *Agrobacterium*, we measured the MDA content in *Agrobacterium* before and after EGCG treatment. The results showed that MDA levels increased with prolonged EGCG treatment and were consistently higher in the EGCG treatment group compared to the control group (Figure 1B). These findings align with those of Li et al. [25], who reported that tea polyphenols damage the cell membrane of *Agrobacterium*. This may be because EGCG’s strong chelating ability stems from the two gallic acid catechol rings, which can bind metal ions such as zinc, copper, and iron. These metals are crucial for bacterial cell wall integrity, enzyme function, and redox reactions [44]. This was further supported by transcriptomic data showing a reduction in the expression of membrane-bound proteins, including *virB4*, *virB7*, and *virB10* (Figure 6).

In eukaryotes, the cell membrane functions as a platform for various cellular processes, including signal transduction and transport. Research has demonstrated that deficiencies in *Agrobacterium* membrane lipids can curtail bacterial motility and virulence, impeding their capacity to infect host cells. Treatment of *Agrobacterium* with EGCG has been demonstrated to result in a significant inhibition of the flagellar synthesis pathway (Figure 5), which may be attributable to membrane damage. We found that 43 genes in the ribosome pathway were significantly inhibited after EGCG treatment for 15 h (Supplementary Table S8A); two oligopeptide transporters in the ABC transport pathway, oppB and oppC, were significantly inhibited after EGCG treatment for 15 h (Supplementary Table S8B). These results prove that EGCG may cause auxotrophic defects in *Agrobacterium*, resulting in the inability of *Agrobacterium* to grow normally, and ultimately making it difficult for *Agrobacterium* to transform plants [32,43]. Furthermore, it has been demonstrated that certain protein kinases present during the cell cycle are capable of regulating mitochondrial oxidative respiration and thereby affecting energy production. Research has demonstrated that tea polyphenols have a detrimental effect on the activity of ATP synthase and can also increase the permeability of bacterial cell membranes [45]. This has been demonstrated to result in ATP leakage. Furthermore, our findings indicate that the assembly of respiratory chain complex IV (GO:0008535) was significantly enriched in the EGCG-treated group (Figure 4), thereby substantiating the substantial impact of EGCG on *Agrobacterium*’s energy production.

Additionally, oxidative stress is known to be a significant limiting factor in *Agrobacterium*-mediated transformation [46]. Therefore, we suggest that EGCG may be a key factor contributing to the recalcitrance of tea plants to *Agrobacterium*-mediated transformation. However, understanding the exact molecular pathways through which EGCG induces oxidative stress and disrupts membrane integrity remains an area that requires further investigation. Such insights could inform the development of strategies to mitigate these inhibitory effects, thereby enhancing transformation efficiency in tea plants.

### 4.2. EGCG Hinders Agrobacterium Biofilm Formation

*Agrobacterium* multiplies by attaching to host cells and secreting a polysaccharide matrix that forms a biofilm, which is critical for successful attachment to plant tissues and effective transformation [12,47]. The high-density microbial community within the biofilm facilitates the expression and function of genes essential for transformation [48]. Flagella play a crucial role in biofilm formation, as they enable bacterial motility and attachment to host surfaces [49].

In this study, transcriptome and qRT-PCR analyses revealed significant differences in the expression of flagellar-synthesis-related genes under EGCG treatment (Figure 3 and Figure S1). GO and KEGG analyses further confirmed the enrichment of the flagellar synthesis pathway (Figure 4 and Figure S2). Flagellar formation involves multiple proteins, including FliE, FlgB, FlgC, FlgF, FlaA, FlaB, FlaC, FlaD, and MotB [50]. Notably, mutations in *flgE* and *motA* have been shown to hinder biofilm formation in *Agrobacterium tumefaciens* C58 [10,51]. Additionally, studies have demonstrated that deletions in *flaA*, *flaB*, and *flaC* inhibit flagellar formation and impair bacterial motility [52], while the absence of *fliN* reduces flagellar rotation and biofilm formation [53,54].

Our results showed that DEGs were significantly enriched in the flagellar synthesis pathway (Figure S2), and EGCG significantly down-regulated the expression of key flagellar synthesis genes (*flaA*, *flaB*, *flaC*, *flaD*, *flgE*, and *motA*) after 2 and 15 h of treatment, with expression levels reaching their lowest at 15 h (Figure 5). This suggests that EGCG impairs *Agrobacterium* flagellar motility and indirectly disrupts biofilm formation, reducing its ability to attach to tea cells. These findings are consistent with previous studies showing that catechins inhibit flagellar-synthesis-related genes (*motY* and *flaC*) in *Vibrio cholerae* [55], and that EGCG inhibits swimming and biofilm formation in *Vibrio mimicus* [56].

The FlgE protein, a subunit of the flagellar hook, is essential for connecting the basal body to filaments and determining bacterial motility [57]. In *P. aeruginosa*, mutations in *flgE* reduce the expression of biofilm-related genes (*pslAB* and *pelAB*) [58]. Zheng et al. [59] found that *flgE* flagellar mutants of *M. tianshanense* had a 41% or 65% reduction in attachment to host cells. Although the role of *flgE* in *Agrobacterium* remains less understood [60], our WGCNA analysis identified *flgE* as a key gene in the brown module (Figure 8A), suggesting that it may be particularly sensitive to EGCG.

In summary, EGCG disrupts *Agrobacteriu* flagellar synthesis and motility, which in turn impairs biofilm formation and reduces bacterial attachment to tea cells. This may explain the observed decrease in *Agrobacterium*-mediated transformation efficiency in tea plants, potentially due to the abundance of phenolic compounds like EGCG. Further research is needed to explore the specific mechanisms by which EGCG affects *flgE* and other flagellar components, as well as its broader implications for genetic transformation efficiency.

### 4.3. EGCG Disrupted the T-DNA Transfer of Agrobacterium 

The virulence level of *Agrobacterium* species plays a key role in plant gene transformation. When plant tissue is damaged, chemical inducers released by the injury activate *Agrobacterium vir* gene expression, which is essential for T-DNA transfer and subsequent gene transformation [61]. Our study revealed that the secretion system pathway of *Agrobacterium* was significantly enriched under EGCG treatment, suggesting that EGCG may disrupt the secretion process of virulence factors (Figure 6, Figure S1, and Figure S2). The type IV secretion system (T4SS) is directly involved in mating-pair formation and the transfer of Ti-plasmid DNA and effector proteins [62,63,64]. Consistent with previous studies showing that catechin inhibits the expression of vir genes (e.g., *virA*, *virB2*, *virD1*, *virD2*, *virD4*, *virF*, *and virK*) in LB medium [41], our results demonstrated that EGCG significantly down-regulated the expression of 11 *virB* T4SS-related genes (*virB1*-*virB11*) and *virD4* (*ATU_RS23875*, *ATU_RS24465*) after 7 and 15 h of treatment (Figure 6). Furthermore, WGCNA analysis identified *virB4* and *virB6* as hub genes in the yellow-green module (Figure 8B). These genes are critical for T-DNA transfer: VirB4 provides the energy required for substrate translocation [65], while VirB6, a multi-layer inner membrane protein, is essential for maintaining VirB5 levels and facilitating substrate transfer [66]. The down-regulation of these genes under EGCG treatment suggests that EGCG may impair T4SS function, thereby hindering T-DNA transfer.

In addition to T4SS-related genes, EGCG also down-regulated the expression of genes involved in protein transport (*secB*, *secDF*, *secG*, *traG*, *tatA*, *tatB*), which are responsible for transporting proteins across the cell membrane (Figure 6) [63,67]. This further supports the hypothesis that EGCG disrupts *Agrobacterium*’s secretion system and protein transport capabilities, contributing to the observed reduction in transformation efficiency.

Quorum sensing (QS) signaling molecules, such as OC8-HSL, play a crucial role in facilitating T-DNA transfer [68]. Under EGCG stress, we observed the up-regulation of *attM* and *traM* genes (Supplementary Table S8B), which encode proteins that degrade QS signaling molecules (AttM) and inactivate the TraR-OC8HSL dimer (TraM) [39,69,70]. These findings suggest that EGCG may further impede T-DNA transfer by diminishing bacterial QS signaling, thereby complicating *Agrobacterium*-mediated transformation of tea leaves.

In summary, our results indicate that EGCG interferes with *Agrobacterium*’s secretion system, protein transport, and quorum sensing, likely contributing to the reduced transformation efficiency observed in *Camellia sinensis* leaves. However, further research is needed to explore whether these effects can be mitigated through chemical inhibitors or genetic modifications in *Agrobacterium*. Such strategies could improve the efficiency of genetic transformation in tea plants, facilitating more effective breeding programs.

## 5. Conclusions

In this study, we investigated the effects of EGCG on *Agrobacterium*-mediated transformation (AMT) efficiency in tea plants. Fluorescence microscopy revealed that 0.4 mg/mL EGCG significantly inhibited transformation efficiency and damaged the *Agrobacterium* cell membrane. Transcriptomic analysis identified two key pathways—flagellar assembly and the secretion system—as potential contributors to the observed reduction in transformation efficiency. We hypothesize that EGCG disrupts biofilm synthesis, impairs the establishment of the secretion system, and hinders *Agrobacterium* attachment to tea plant cells, thereby inhibiting T-DNA transfer. Through weighted gene co-expression network analysis (WGCNA), we identified *flgE*, *virB4*, and *virB6* as key genes that may play critical roles in overcoming the resistance mechanisms of tea plants to AMT. These findings provide new insights into the molecular mechanisms underlying EGCG’s inhibitory effects on *Agrobacterium* transformation and highlight potential targets for improving genetic transformation efficiency in tea plants.

## Data Availability

The datasets generated and/or analyzed during the current study are available in the NCBI repository, Bio project PRJNA1067299.

## Supplementary Materials

The following supporting information can be downloaded at: https://www.mdpi.com/article/10.3390/cimb47030178/s1.
