# Supplementary figures and images for: Transcriptomic Analysis of the Negative Effect of Epigallocatechin-3-Gallate from Tea Plant (Camellia sinensis) on Agrobacterium-Mediated Transformation Efficiency
Source: Curr Issues Mol Biol. 2025 Mar 8;47(3):178. doi: 10.3390/cimb47030178 (PMC11941606; doi:10.3390/cimb47030178)

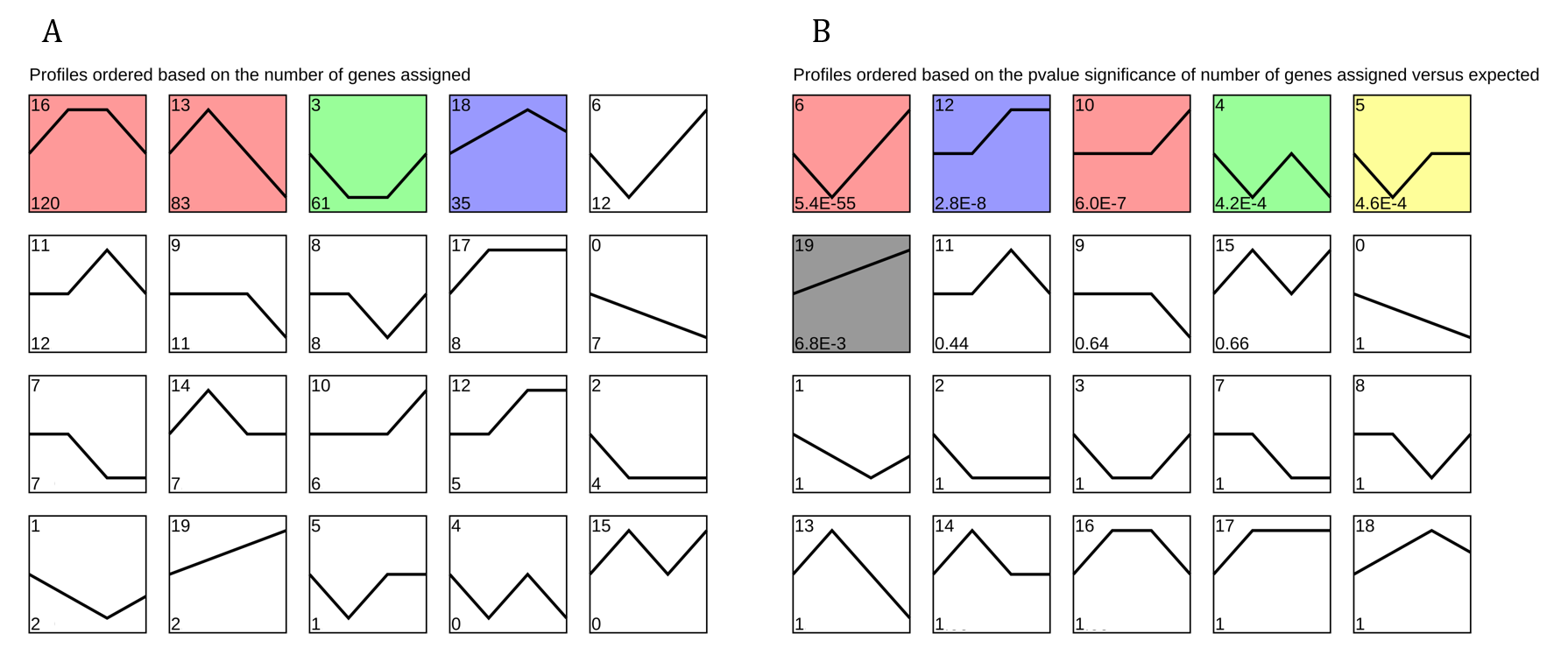

Supplement: Supplementary file 1 [file cimb-47-00178-s001.zip › cimb-3453937-supplementary/Figure/Fig S1.tif]

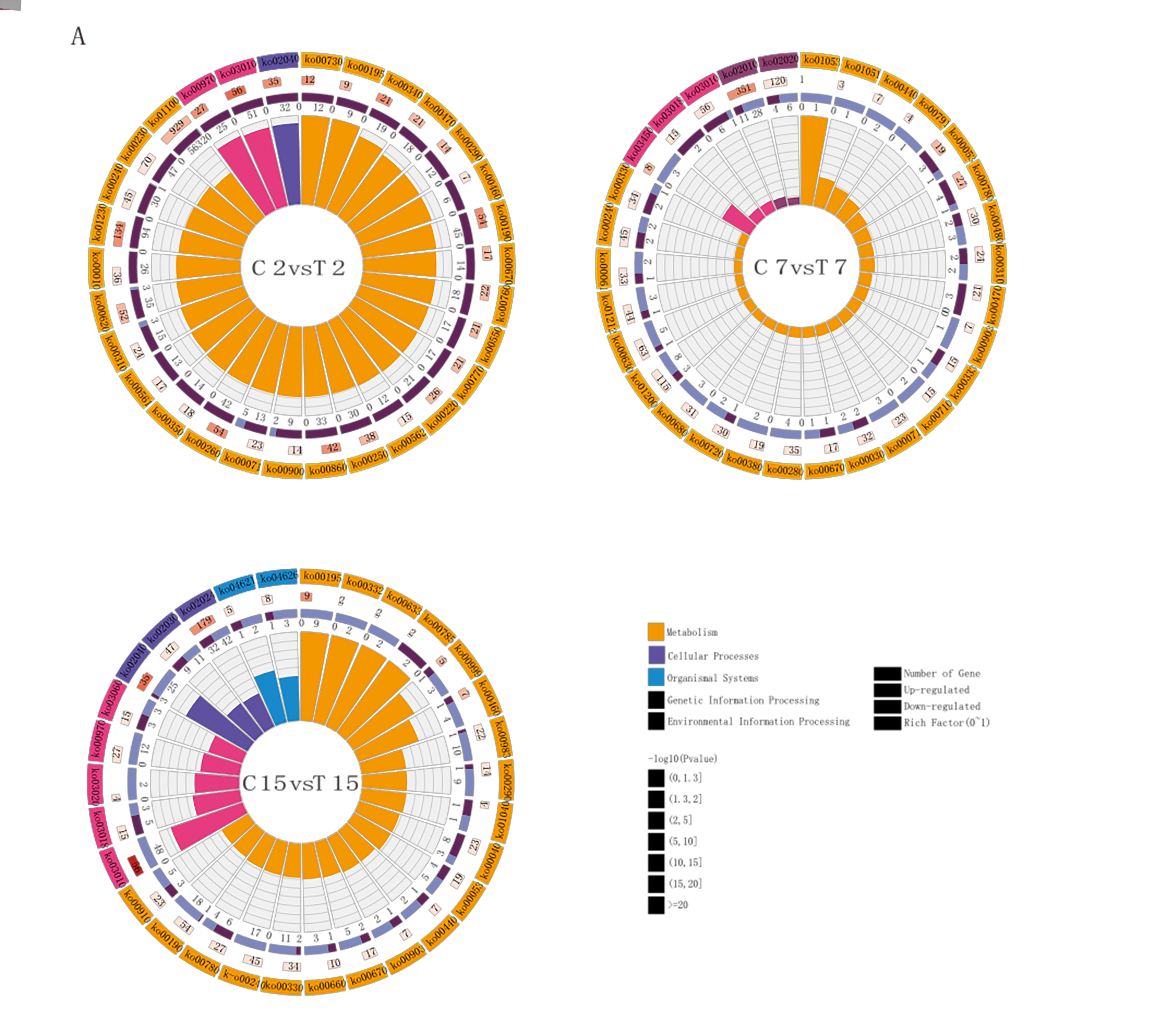

Supplement: Supplementary file 1 [file cimb-47-00178-s001.zip › cimb-3453937-supplementary/Figure/Fig S2.tif]
